# The SLaM Brain Health Clinic: remote biomarker enhanced memory clinic for people with mild cognitive impairment within a National Health Service mental health trust

**DOI:** 10.1192/bjo.2024.829

**Published:** 2024-12-19

**Authors:** Ashwin V. Venkataraman, Pooja Kandangwa, Roos Lemmen, Rutvi Savla, Mazda Beigi, Devon Hammond, Daniel Harwood, Justin Sauer, Latha Velayudhan, Clive Ballard, Anna-Katharine Brem, Chris Kalafatis, Dag Aarsland

**Affiliations:** Centre for Healthy Brain Ageing, Institute of Psychiatry, Psychology & Neuroscience, King's College London, UK; Centre for Neuroimaging Sciences, Institute of Psychiatry, Psychology & Neuroscience, King's College London, UK; South London and Maudsley NHS Foundation Trust, London, UK; College of Medicine and Health, University of Exeter, UK; University Hospital of Old Age Psychiatry, University of Bern, Switzerland; Centre for Age-Related Research, Stavanger University Hospital, Norway

**Keywords:** Remote brain health clinic, biomarkers, old age psychiatry, mild cognitive impairment, dementia

## Abstract

**Background:**

The novel South London and Maudsley Brain Health Clinic (SLaM BHC) leverages advances in remote consultations and biomarkers to provide a timely, cost-efficient and accurate diagnosis in mild cognitive impairment (MCI).

**Aims:**

To describe the organisation, patient cohort and acceptability of the remote diagnostic and interventional procedures.

**Method:**

We describe the recruitment, consultation set-up, the clinical and biomarker programme, and the two online group interventions for cognitive wellbeing and lifestyle change. We evaluate the acceptability of the remote consultations, lumbar puncture, saliva genotyping, and remote cognitive and functional assessments.

**Results:**

We present the results of the first 68 (mean age 73, 55% female, 43% minoritised ethnicity) of 146 people who enrolled for full remote clinical, cognitive, genetic, cerebrospinal fluid and neuroimaging phenotyping. A total of 86% were very satisfied/satisfied with the remote service. In all, 67% consented to lumbar puncture, and 95% of those were very satisfied, all having no significant complications. A total of 93% found taking saliva genotyping very easy/easy, and 93% found the cognitive assessments instructions clear. In all, 98% were satisfied with the Cognitive Wellbeing Group, and 90% of goals were achieved in the Lifestyle Intervention Group.

**Conclusions:**

The SLaM BHC provides a highly acceptable and safe clinical model for remote assessments and lumbar punctures in a representative, ethnically diverse population. This allows early and accurate diagnosis of Alzheimer's disease, differentiation from other MCI causes and targets modifiable risk factors. This is crucial for future disease modification, ensuring equitable access to research, and provides precise, timely and cost-efficient diagnoses in UK mental health services.

Dementia, of which the largest cause is Alzheimer's disease, affects 50 million people globally with a predicted threefold increase by 2050. In the UK, revised increased estimates suggest 1.7 million people will have dementia by 2040.^1^ An even higher number of people have mild cognitive impairment (MCI), and many of them are in the prodromal stage of Alzheimer's disease.^2^ The emergence of new disease-modifying therapies for Alzheimer's disease is a huge opportunity, but also a challenge.^3–5^ A total of 30 200 people per year are expected to be eligible for disease-modifying monoclonal antibody therapies for Alzheimer's disease in the UK.^6^ Waiting times from referral to diagnosis are 13 weeks,^7^ with expected waiting times for access forecast to be 56 months in 2023, increasing to 129 months in 2029, hence the need for rapid change and innovative approaches in this field.^8,9^

There are now rapid advances in digital, imaging and molecular biomarkers of Alzheimer's disease^10–14^ and remote assessment opportunities,^15,16^ alongside the emergence of new therapies and knowledge of targeting modifiable risk factors.^17^ Early accurate aetiological diagnosis of Alzheimer's disease is crucial to enable adequate treatment and is in line with public attitudes,^18^ but the uptake of the diagnostic biomarkers is extremely low in some countries, including the UK.^7^ There is therefore a clear need for memory services to rapidly adapt to this new landscape for greater patient benefit, and to match the molecular and digital biomarker developments globally in this field.^19^ Notably there is a huge gap that exists between demand and assessment – 99% of people with MCI never receive a diagnosis and are not referred to memory clinics; this must increase.^20^ These increases must also address disparities across ethnicity and socioeconomic status. This is particularly important in mental health trusts in the UK who see 92% of people with memory complaints, with the remainder seen by geriatrics and neurology.^7^

The South London and Maudsley Brain Health Clinic (SLaM BHC) is an innovative, remote service within a mental health trust that leverages advances in the accessibility of remote consultations combined with detailed biomarker assessment, with the aim of addressing these new challenges for the healthcare system. Here we describe the organisation, the diagnostic and intervention procedures, and the interventional groups. We describe the key characteristics of the first 146 referrals to the SLaM BHC, and the experience, feasibility and acceptability for those who signed up for the linked BHC research project.

## Method

### Recruitment and participants

The South London and Maudsley National Health Service (NHS) Foundation Trust in the UK covers a catchment population of over 1.3 million people across four London boroughs. Within this trust, referrals were made to the BHC via three memory services (Croydon, Lewisham and the combined service for Southwark and Lambeth) after an initial clinical assessment and diagnosis by either a psychiatrist or a nurse specialist, with cognitive testing, routine dementia exclusion bloods and possible additional brain imaging.

The SLaM BHC research protocol was approved under the Research Ethics Committee (REC) 22/SC/0109 (South Central – Berkshire B) and enabled the use of cerebrospinal fluid (CSF), and genetic and remote cognitive and functional biomarkers for all research participants. Out of the total of 5,751 referrals for all cognitive problems to the three memory services, 146 referrals were accepted to the SLaM BHC and fulfilled the inclusion/exclusion criteria below. The clinic began taking referrals from three SLaM memory services in October 2021 and closed to referrals in July 2023, and continued seeing the research participants. Up to 1 January 2024, 68 have consented and completed the initial assessment of the SLaM BHC research project, 40 participants will be approached during 2024, and 45 declined or were ineligible and were of similar demographics to those that consented. People who did not consent to the research project were offered treatment as usual, including longitudinal clinician evaluation and the intervention groups as shown below.

**Fig. 1:**
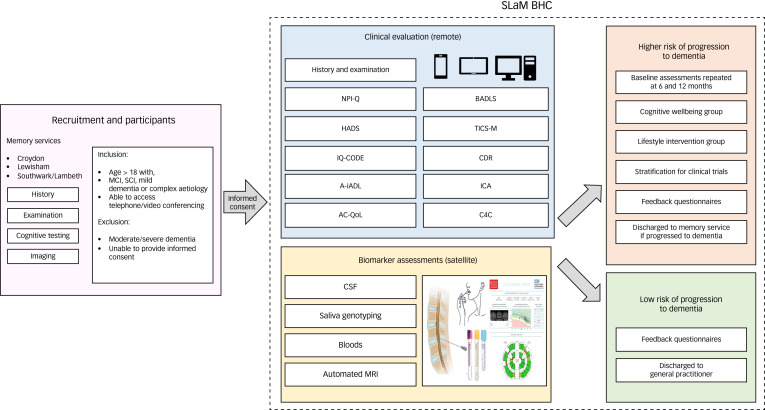
Overview of the South London and Maudsley Brain Health Clinic (SLaM BHC) showing the recruitment and participants from three memory services, with inclusion criteria including those with mild cognitive impairment (MCI) and subjective cognitive impairment (SCI), or mild dementia of uncertain or complex aetiology and exclusion criteria following referral to the SLaM BHC. Clinical evaluation comprised history and examination, neuropsychiatric inventory (NPI-Q), the hospital anxiety and depression scale (HADS), informant questionnaire on cognitive decline in the elderly (IQ-CODE), Amsterdam Instrumental Activities of Daily Living (A-iADL) functional assessment, adult carer quality of life questionnaire (AC-QoL), telephone interview for cognitive status for memory (TICS-M), clinical disease rating (CDR), integrated cognitive assessment (ICA) and patient reported experience and outcome measures, Bristol activities of daily living scale (BADLS), and assessment for consent for contact for research (C4C). Satellite procedures for biomarker assessments included lumbar puncture for cerebrospinal fluid (CSF), saliva genotyping, bloods and automated magnetic resonance imaging (MRI). Following this information, individuals were stratified into higher risk of progression to dementia or lower risk of progression to dementia with listed outcomes below, with all followed up after 6 and 12 months under the research component.

Inclusion criteria for the SLaM BHC and the research project were people referred by SLaM memory clinics either with a formal diagnosis of MCI, subjective cognitive impairment or mild dementia when the case was aetiologically complex. Additional inclusion criteria were the ability to access the clinic via telephone or video conferencing. Exclusion criteria were a diagnosis of moderate–severe dementia, or those unwilling or unable to provide written consent because of lacking capacity, for example. All medications and treatments were permitted concurrently while engaging in this study and were flagged at the time of referral if affecting cognition. Figure 1 below shows an overview of the SLaM BHC.

### Consultation setup

All patients seen in the BHC underwent remote assessments using virtual conferencing (via Microsoft Teams and with appropriate help from a caregiver/family member when needed) with a psychiatrist or an experienced nurse clinician, with opportunities for support from the SLaM Digital inclusion team.^21^ In some cases only telephone assessment was possible. Remote clinical evaluation and satellite biomarker assessments were performed, with individual feedback to participants and families via telephone/virtual conferencing following consensus diagnosis of the stage and aetiology in a virtual multidisciplinary team.

### Clinical evaluation programme

The current remote baseline assessment protocol included a detailed patient history, the Telephone Interview for Cognitive Status (TICS-M v39) consisting of 13 items,^22^ the Clinical Disease Rating (CDR) obtained through semi-structured interviews of participants and informants rated across six domains of cognitive functioning,^23^ the neuropsychiatric inventory (NPI),^24^ the Hospital Anxiety and Depression Scale (HADS) – a self assessment scale,^25^ Consent for Contact for research (C4C) – the SLaM research register consent,^26^ and patient-reported experience and outcome measures. The Informant Questionnaire on Cognitive Decline in the Elderly (IQ-CODE),^27^ the digital version of the Amsterdam Instrumental Activities of Daily Living (A-iADL) functional assessment,^28^ Bristol Activities of Daily Living Scale (BADLS)^29^ and computerised cognitive assessments were emailed to participants to obtain information on daily living activities. The Integrated Cognitive Assessment (ICA) is a 5-min computerised cognitive test based on a rapid categorisation task that employs an artificial intelligence model to improve its accuracy in detecting cognitive impairment;^30^ it is self-administered and independent of language.^31,32^ We will focus on the feasibility aspects and will not present the individual results of these tests.

### Biomarker programme

The BHC research project biomarker programme for those consenting included CSF for Alzheimer's disease markers and saliva for genetics. In addition, bloods for Alzheimer's disease markers and magnetic resonance imaging (MRI) are included but not presented here. Lumbar puncture was performed by a neurologist on a pay-for-service basis at the Biomedical Research Centre (BRC) Clinical Research Facility. CSF was collected in polypropylene tubes then centrifuged within 1 h of collection at 2000 xg, room temperature for 10 min, transferred to new polypropylene tubes, transferred to barcoded cryovials (up to 20 aliquots at least 450 μL each) with log barcodes of cryovials used for each subject and stored in a –80°C freezer in the Department of Old Age Psychiatry, Institute of Psychiatry, Psychology and Neuroscience (IoPPN), Kings College London (KCL) laboratory. CSF analysis was performed using the ElectroChemiLuminescence Immunoassay Instrument: Cobas® 6000 analyser series. The assays are Elecsys® β-Amyloid(1–42) CSF, Elecsys® Phospho-Tau (181P) CSF & Elecsys® Total-Tau CSF) at Affinity Biomarker Labs with predefined cut-offs based on the Elecsys® assays. Saliva samples were analysed by Cytox Group Limited, employing a polygenic risk scoring algorithm, genoSCORE™LAB, including apolipoprotein E genotyping, to identify those at highest genetic risk of Alzheimer's disease using genetic data from the saliva sample.^33^ Additionally, participants provided a blood sample that was immediately centrifuged, with plasma extracted and stored in a -80°C freezer in the Department of Old Age Psychiatry, IoPPN, KCL laboratory for use in future diagnostic dementia biomarker studies.^14,34^ Automated volumetric MRI analysis pipelines extracted regional volumes compared with normative populations using a geodesic information flow algorithm in addition to training on the SLaM Image Bank,^12,35,36^ following routine structural MRI acquisition as per dementia scanning protocols at the Centre for Neuroimaging Sciences, King's College London, including at least T1-weighted structural MRI (3D Coronal MPRAGE), T2-weighted structural MRI (2D Axial T2, 2D Axial FLAIR) and diffusion tensor imaging (15-direction DTI).

Following this information, individuals were stratified by the assessing clinician into higher or lower risk of progression to dementia. Those at higher risk of progression were offered clinical trials or non-pharmacological studies based on both individual preference and trial eligibility criteria. Individuals who were diagnosed with dementia while under follow-up at the SLaM BHC were transferred back to the memory service. Patients who were not diagnosed with a neurodegenerative disease following assessment by the SLaM BHC were discharged to their general practitioner (GP). All were followed up under the research component at the 6- and 12-month time point, regardless of risk.

### Two intervention groups

The SLaM BHC developed two fully remote online psychological intervention groups as part of the clinical procedure, to which all participants were invited. The first was the Cognitive Wellbeing Group which focused on psychoeducation on brain anatomy, cognition, MCI, dementia and psychological concepts with strategies to manage memory and mood-related difficulties. The second group was the Lifestyle Intervention Group which focused on dementia prevention and the impact of lifestyle factors on cognition and the potential for lifestyle changes, including goal setting, physical health, physical activity, nutrition, sleep, keeping one's mind active, social activity and compensatory techniques for memory. Both groups consisted of six to eight people, ran over eight 1h sessions per round and were led by two clinicians, one psychologist and one psychology assistant.

### Feasibility and acceptability assessments

Participant feedback was analysed with a semi-structured interview outcome. Feedback on the clinic and individual virtual technologies was given specifically for lumbar punctures, genoscore, the patient-reported experience and outcome measures, digital biomarkers (Amsterdam iADL, ICA) and feedback questionnaires, alongside semi-structured interviews for the groups.

## Results

### Cohort and feasibility

As seen in Table 1, the full cohort is fairly representative for an NHS memory clinic^18^ with regard to age (mean 75 years) and gender (64% female) with a higher percentage of ethnic diversity (58% White, 42% minoritised ethnicity, compared with 87% White nationally^7^) and lower education (53% having secondary school or less). Of 135 participants the majority, 73%, were able to complete the virtual assessment, whereas 27% could do telephone assessment only, with no significant inequality differences between groups. Median wait time from referral to assessment was 44 days. The majority of participants were able to complete the full clinical cognitive assessments and the biomarker acquisition procedures.

**Table 1:** Demographic and clinical characteristics of the cohort stratified by number of patients completing the various biomarker procedures

	Total cohort	Consented to research study	Consented to lumbar puncture	Cognitive Wellbeing Group	Lifestyle Intervention Group
*N*	146	68	45	25	4
Age (mean, range)	75 (53–96)	73 (53–89)	71 (53–89)	73 (53–89)	59 (53–64)
Gender (*n*, %)					
Female	94 (64)	37 (55)	26 (58)	17 (68)	4 (100)
Male	52 (36)	31 (45)	19 (42)	8 (32)	0
Ethnicity (*n*, %)					
Asian	15 (10)	10 (15)	7 (15)	3 (12)	0
Black	33 (23)	14 (21)	8 (18)	7 (28)	2 (50)
Mixed ethnicity	9 (6)	1 (1)	0	1 (4)	0
White	85 (58)	39 (57)	27 (60)	14 (56)	2 (50)
Other ethnic group	4 (3)	4 (6)	3 (7)	0	0
Highest level of education (*n*, %)					
Primary school	12 (8)	2 (3)	1 (2)	0	0
Secondary school	65 (45)	35 (51)	27 (60)	13 (52)	3 (75)
Bachelor's degree or equivalent	30 (21)	17 (25)	9 (20)	8 (32)	0
Master's degree or equivalent	3 (2)	6 (9)	5 (11)	1 (4)	1 (25)
PhD, doctorate or equivalent	12 (8)	7 (10)	3 (7)	2 (8)	0
Missing	24 (16)	1 (2)	0	1 (4)	0
Cognitive stage (*n*, %)					
Mild cognitive impairment	114 (78)	56 (82)			
Dementia	27 (19)	10 (15)			
Other	5 (3)	2 (3)			

Of the 68 who consented to the research protocol, 45 (66%) also consented to lumbar puncture. They had similar demographics to the full cohort. Of the 45 available, 26 participants have had an lumbar puncture to date with three failed lumbar punctures (two because of lumbar degeneration and one because of patient girth), and the remainder pending or contraindicated because of medications. A total of 15/23 available results (65%) had an Aβ42 value below the cut-off with 55% having a positive pTau/Aβ42 ratio. The average turnaround time from CSF sample taken to result was 1 day (range 0–4 days).The median time from consent to CSF results back was 60 days. Of the available genoSCORE results for 35 participants, 18 (52%) had at least one e4 allele and 17 (48%) had no e4 allele, with 19 (54%) having a high risk of progression to Alzheimer's disease from the polygenic risk score, 6 (17%) having a medium risk and 10 (29%) having a low risk. Average digital cognitive score results at baseline were TICS-M 22/39, CDR global score 0.5, CDR sum of boxes 2.75, ICA 54 (speed 67%, accuracy 76%, probability of impairment 71%), IQ-CODE 3.5, A-iADLs 13.95 and NPI 9.21. The average turnaround of genoscore results was 72 days (range 9–229 days). In all, 13% had medial temporal lobe atrophy, 88% had a general cerebral atrophy score of 1 and 12% had a score of 2. A total of 25 participants attended the Cognitive Wellbeing Group and 4 attended the Lifestyle Intervention Group.

A total of 30% of participants were not diagnosed with a neurodegenerative disease following assessment and were discharged to their GP. The cognitive stage of the full cohort showed that 114 (78%) had MCI and 27 (19%) fulfilled criteria for dementia, with similar proportions in the research cohort with 56 (82%) having MCI, and 10 (15%) having dementia at the last recorded time point (Table 1).

### Feedback

Feedback from 43 participants for the overall remote BHC procedures was available and representative of the total and research cohort (mean age 74, 53% female, 52% minoritised ethnicity). In all, 17 (40%) found technologies for assessments and appointments either very easy or easy, 20 (47%) were neutral, 3 (7%) found them difficult/very difficult and 3 (7%) did not respond. A total of 26 (60%) would recommend this to friends and family, 3 (7%) would not recommend it and 11 (26%) did not respond. In all, 30 (70%) were able to contact a team clinician when needed, 2 (5%) were unable and 7 (16%) did not respond. A total of 37 (86%) were either very satisfied or satisfied with the overall service, 4 (9%) were neutral, 0 were dissatisfied and 2 (5%) did not respond. Further details of feedback are provided in Appendix Table 1.

For the lumbar puncture procedure, 20 of 21 (95%) respondents were very satisfied and one (5%) was satisfied. Five had concerns before the procedure, and all responded that they had had the opportunity to ask questions, thought the information sheet was helpful and were able to contact a clinician when they needed; only one (5%) experienced complications (‘anxiety about the results’) whereas 20 (95%) reported no complications (Appendix Table 2). Of the 45 genoscore feedback results, 42 (93%) found taking the saliva sample very easy or easy, and 100% found the instructions clear, with 41 (91%) stating after taking it that they would not have preferred doing this in clinic (Appendix Table 3).

A total of 45 participants completed the ICA feedback, with 42 (93%) finding the instructions clear and 31 (69%) not requiring support when completing the test (Appendix Table 4). Feedback on the intervention groups was available for 15 participants. As seen in Appendix Table 5, the feedback was very positive, with 14 (98%) finding the group very helpful, 1 (5%) neutral and none unhelpful. All felt the groups helped them better understand both MCI and the impact of mental health on cognition. They were representative of the demographics of the whole cohort (Table 1). In the Lifestyle Intervention Group, 90% of goals that were set were achieved successfully. Semi-structured interview and feedback questionnaire results are shown in Appendix Table 6.

## Discussion

The SLaM BHC successfully provides early and accurate diagnoses of Alzheimer's disease in people with MCI, along with a safe and acceptable care model for various remote clinical, cognitive and biomarker assessments within an NHS mental health memory setting. This is crucial in preparing for disease modification, stratifying risk and enhancing clinical research access, with the opportunity for secondary prevention of cognitive decline.

The clinic has a number of strengths and demonstrated that it is now possible to provide remote clinical assessments for people, with high acceptability and very positive participant feedback. We also show that satellite biomarker evaluations for CSF, genotyping, bloods and neuroimaging are not only possible but highly acceptable, with relatively fast turnaround times to the results once taken and without any adverse events. Furthermore, we show this is possible in an ethnically diverse and representative cohort in South London with a higher proportion from less educated and more deprived backgrounds. We were able to show early and accurate diagnoses of Alzheimer's disease in half of participants, with a third being discharged to the GP with no evidence of neurodegenerative disease. Finally we were able to implement effective secondary prevention interventions from the Cognitive Wellbeing Group and Lifestyle Intervention Group for elderly people in the comfort of their own homes.

We know that older people are at higher risk of reduced physical and social activity, loneliness and depression, which are all factors associated with more rapid cognitive and functional decline.^37^ Recent technological advances of remote memory assessments can provide an opportunity to re-evaluate how existing methods can be adapted for remote assessment and how digital technology can be used to automate cognitive assessments and data collection.^15,16^ Advantages include that they avoid travel fatigue, have adjustable volume/screen size for better hearing and lip-reading, have cognitive tests magnified and also allow visualisation of the home environment, similar to home visits. This approach reduces carbon emissions, clinical infrastructure costs and non-attendance rates, addressing some hurdles of face-to-face consultations. In addition, remote biomarkers provide the opportunity to further increase capacity and meet unmet demand. This is particularly important, given that most people with MCI never receive a specific diagnosis, and therefore there would need to be a necessary shift for accurate primary care-based Alzheimer's disease diagnoses using new methods to facilitate this.^38^

The main limitation of the SLaM BHC to date is the small sample size. However, this is mainly because of limited capacity to include all eligible and consenting participants. In addition those referred to the service were potentially those more likely to engage in the programme. Importantly, the participants in the research component did not differ from the overall referral cohort regarding age, ethnicity and education, showing that this research component is representative. Although we have shown the majority of this cohort were able to perform the procedures, a considerable proportion did not complete the digital set-up; digital exclusion is therefore a critical issue. This is expected to gradually become a smaller problem as digital competence increases. Mitigating strategies for people who need digital support, such as that available from the SLaM Digital Inclusion Team,^21^ would help with digital exclusion; however, the support was only available for a limited time period.

Future plans for the SLaM BHC are related to scaling up assessments across other memory clinics in South London more widely and clinical workflows that are focused on the importance of actionable guidance towards prevention.^38^ The potential of using remote assessments and risk reduction that can be done in people's homes and funnelled to GPs such as in the prediction of Alzheimer's disease using an AI driven screening platform (PREDICTOM) study^39^ and the Alzheimer's disease real-world implementation, deployment, and validation of early detection tools and lifestyle enhancement (AD-RIDDLE)^40^ have the potential to improve the precision of referrals.^41^ Future blood-based markers^14^ and novel cognitive training games with additional clinical decision support tools may also be utilised in this remote diagnostic and interventional pathway.

We have successfully shown that the remote SLaM BHC can provide an early and accurate diagnosis of Alzheimer's disease in people with MCI in an NHS mental health trust in a diverse and representative population. It also presents an opportunity for addressing modifiable risk factors, gives safe and appropriate care for people undergoing lumbar puncture and genotyping, and provides an acceptable model for remote assessments to increase the diagnostic capacity to meet unmet demand. This will be crucial in preparing for the prospect of disease modification and enhancing access disparities to clinical research and trials, alongside providing more precise diagnoses to patients and families.

## Data Availability

The data that support the findings of this study are available from the corresponding author, A.V.V., upon reasonable request.

## Appendix

**Appendix Table 1:** Feedback on remote assessments in the South London and Maudsley Brain Health Clinic

Questions	*N* = 43
	*n* (%)
**How did you find using the technologies for your assessments and appointments?**	
Easy/Very easy	17 (40)
Neither	20 (47)
Difficult/Very difficult	3 (7)
Did not respond	3 (7)
**Having completed the online assessments, would you recommend them to your friends and family?**	
Yes	26 (60)
No	3 (7)
Not applicable	3 (7)
Did not respond	11 (26)
**Were you able to contact a team clinician when you needed to?**	
Yes	30 (70)
No	2 (5)
Not applicable	4 (9)
Did not respond	7 (16)
**How satisfied are you overall with the service you have received?**	
Satisfied/Very satisfied	37 (86)
Neither satisfied or dissatisfied	4 (9)
Dissatisfied/Very dissatisfied	0
Did not respond	2 (5)

**Appendix Table 2:** Lumbar puncture feedback

Questions	*N* = 21
	*n* (%)
**How satisfied were you with the procedure?**	
Very satisfied	20 (95)
Satisfied	1 (5)
Neither satisfied/dissatisfied	0 (0)
Dissatisfied	0 (0)
**Did you have any concerns about the procedure?**	
Yes	5 (24)
No	16 (76)
**Did you have an opportunity to ask questions?**	
a) Yes	21 (100)
b) No	0 (0)
**Was the information sheet helpful?**	
Yes	21 (100)
No	0 (0)
**Did you experience any complications after the procedure?**	
Yes	1 (5) ‘felt anxious about results’
No	20 (95)
**Were you able to contact a clinician if needed?**	
Yes	21 (100)
No	0 (0)

**Appendix Table 3:** Genoscore feedback

Questions	*N* = 45 *n* (%)
**How did you find taking the sample?**	
Very easy	17 (37)
Easy	25 (55)
Neither difficult nor easy	3 (7)
Difficult	0 (0)
Very difficult	0 (0)
**How did you find sending the sample?**	
Very easy	20 (44)
Easy	20 (44)
Neither difficult nor easy	4 (9)
Difficult	1 (2)
Very difficult	0 (0)
**Were the instructions clear?**	
Yes	45 (100)
No	0 (0)
**4) Having taken the sample yourself, would you have preferred to have completed it in a clinic?**	
Yes	4 (9)
No	41 (91)
**Did you require any support when taking the sample?**	
Yes	15 (33)
No	30 (67)
**If you needed to, were you able to contact a clinician for support?**	
Yes	25 (56)
No	1 (2)
Not applicable	19 (42)

**Appendix Table 4:** Integrated cognitive assessment (ICA) feedback

Questions	*N* = 45 *n* (%)
**How did you find using the ICA-Comp?**	
Very easy	3 (7)
Easy	10 (22)
Neither difficult nor easy	13 (29)
Difficult	9 (20)
Very difficult	10 (22)
**Were the instructions clear?**	
Yes	42 (93)
No	3 (7)
**Did you require any support when completing the test?**	
Yes	14 (31)
No	31 (69)

**Appendix Table 5:** Cognitive Well being Group feedback, with quantified response on a scale of 1–10 (with 10 reflecting a more positive response)

Questions	*N* = 15 *n* (%)
**How did you find the group?**	
Very unhelpful (<5)	0 (0)
Neutral (5)	1 (7)
Very helpful (>5)	14 (93)
**Would you want more groups like this one?**	
Strongly disagree (<5)	0 (0)
Neutral (5)	1 (7)
Strongly agree (>5)	14 (93)
**Have the groups helped you better understand mild cognitive impairment?**	
Not at all (<5)	0 (0)
Neutral (5)	0 (0)
Very much improved (>5)	15 (100)
**Did the groups help you better understand the impact of mental health on cognition?**	
Not at all (<5)	0 (0)
Neutral (5)	0 (0)
Very much (>5)	15 (100)

**Appendix Table 6:** Semi-structured interview and feedback questionnaire results

Positive feedback examples: ‘I am fully satisfied with the service provided. In fact I have recommended it to friends who have the same problems like me.’ ‘The team seem very friendly.’ ‘Enjoyed their detailed explanation of their diagnosis and the state of affairs.’ ‘Clinician explained unfamiliar and complicated concepts very clearly.’ ‘Professional service made me feel confident.’ ‘Very helpful, considered, took the time to explain and give me useful tips to keep my brain active.’ ‘Extremely friendly and caring staff. Very clear instructions. Lots of patience shown when the video call was not connecting initially. Clear debrief and follow-up steps.’ ‘Very good staff!’ ‘My heartfelt thanks!’ ‘Excellent service was received. You are doing a good job helping people in this field.’ ‘You notified me I could start using the new Brain Training Section. I found that very interesting and enjoyable.’
‘Once we managed to get through it was very easy and straightforward.’ ‘The 8 virtual meetings that were arranged were extremely helpful and I subsequently received notes on the topics discussed. I chose to take part in tests for the Research Group and once again the staff were exceptional. I imagine that in the current economic climate your scope to expand the service will be limited. I was lucky that my GP referred me to the clinic and I have provided very positive feedback to him.’ Feedback regarding improvement/next steps examples: ‘Wanted more explanation on how pathology affects the brain.’ ‘It should continue to be available to for people like myself with memory problems.’ ‘What did not work well? Nothing, although given the choice, I would prefer face to face.’ ‘Remote delivery assumes the patient has some understanding of the necessary technology and home-based service to facilitate such. In our case my mother has neither broadband nor a suitably strong 4G mobile coverage in the area she lives in. It was necessary for me to drive her to a location where there was suitable 4G mobile coverage. Overall I'm satisfied with the service and BHC have on offer and the ongoing research/development openings being offered.’ ‘Make the forms easier to understand!’
